# Transitioning Competency-Based Communication Assessments to the Online Platform: Examples and Student Outcomes

**DOI:** 10.3390/pharmacy9010052

**Published:** 2021-03-05

**Authors:** Sarah Scoular, Ashley Huntsberry, Toral Patel, Sara Wettergreen, Jason M. Brunner

**Affiliations:** Department of Clinical Pharmacy, Skaggs School of Pharmacy and Pharmaceutical Sciences, University of Colorado Anschutz Medical Campus, Aurora, CO 80045, USA; ashley.huntsberry@cuanschutz.edu (A.H.); toral.patel@cuanschutz.edu (T.P.); sara.wettergreen@cuanschutz.edu (S.W.); jason.brunner@cuanschutz.edu (J.M.B.)

**Keywords:** pharmacy education, OSCE, virtual, simulation, global, technology, pharmacy learning, educational strategies, COVID-19, remote learning, online education, communication, assessment

## Abstract

In light of the COVID-19 pandemic, pharmacy education has shifted from in-person teaching and assessments to the virtual environment. Many education programs had previously adopted objective structured clinical examinations (OSCEs) to assess communication abilities in-person with standardized patients (SPs). However, comparative student outcome data between virtual and in-person methods as well as guidance on how to conduct communication-based OSCEs in the virtual environment is lacking. The University of Colorado Skaggs School of Pharmacy and Pharmaceutical Sciences (SSPPS) describes its methods of conducting two types of communication-based OSCEs (patient counseling and gathering a medical history). Student performance data from the two virtual OSCEs in 2020 was compared to results from two 2019 in-person OSCEs using Mann Whitney U Tests. The 2020 cohort scored significantly higher than the 2019 cohort in all variables (i.e., using effective verbal and non-verbal communication, using patient friendly education, organizing the encounter, demonstrating empathy, establishing trust, professionalism) and in overall score. However, the effect size for these findings indicate the differences between performances are generally small and more likely due to changes in grading patterns due to the pandemic.

## 1. Introduction

The pharmacy profession has been transitioning from a focus on medication preparation and dispensing roles to patient-centered clinical practice. During this evolution, objective structured clinical examinations (OSCEs) have been increasingly utilized within pharmacy education to assess clinical skills and communication abilities [1,2]. The OSCE is a helpful method that allows for assessment of multiple skill sets as learners complete simulated professional tasks [3]. Most often, OSCEs in pharmacy education are completed in-person with use of standardized patients (SPs) [4]. However, the COVID-19 pandemic quickly necessitated a shift to virtual delivery of OSCEs. While the communications curricula at the University of Colorado Skaggs School of Pharmacy and Pharmaceutical Sciences (SSPPS) previously prepared students for some aspects of telephonic-based care, delivery through virtual visit methods (patient visits conducted over video technology) were not previously assessed. 

SSPPS has utilized OSCEs within its communication courses for 30 years. In 2019, a high-stakes, cumulative, summative assessment incorporating an OSCE component was integrated into the curriculum. This included an assessment of ability to collect a medical history from a patient, and was not part of a course. This will be referred to throughout this study as the Cumulative OSCE. An additional assessment, which will be referred to as the Patient-Centered Communication Course 2 (PCC2) Patient Counseling OSCE, assessed students on counseling a patient on two prescription products with unique dosage formulations. The COVID-19 pandemic prompted a swift shift of both assessments to the virtual setting via Zoom (Zoom Video Conferencing 2020). Student performance on these two different skills-based communication assessments completed virtually in 2020 were compared to student performance on the same assessments completed in-person one year prior. The University of Florida College of Pharmacy recently described implementation of an OSCE in their PharmD program, however this description did not expound upon virtual methods for delivery [5]. Due to the lack of overall guidance on methods for implementing pharmacy based communication OSCEs in the virtual environment, we will also discuss procedures and recommendations for executing a successful virtual OSCE. 

## 2. Materials and Methods

The PCC2 course occurs in the second semester of the first-year curriculum. This assessment was the last of four OSCEs within the course, and thus was used as a sample to compare in-person and online modalities of OSCEs. Student data from the 2019 (in-person) and 2020 (online) cohort were compared to assess differences in performance. For this assessment, students were required to counsel a SP on two prescription products with unique dosage forms (e.g., inhalers). Students’ communication skills were graded by SPs utilizing a standardized rubric focused on verbal and non-verbal communication skills as well as establishing trust, providing patient friendly education and organization of the encounter. Due to the remote nature of the 2020 assessment, product information handouts were utilized as a visual supplement during the counseling session rather than the demo inhalers used during the 2019 in-person assessment. All other procedures remained the same between the two cohorts.

A second sample was collected from the cumulative OSCE comparing the same 2019 (in-person) and 2020 (online) cohorts. The end-of-year cumulative OSCE was implemented in the curriculum in 2018 to promote retention and integration of the curriculum and assure competency before progression to the second year. The OSCE consists of a patient counseling interaction utilizing a SP, specifically the task of collecting a medical history from the patient. Students’ communication skills were graded by SPs using a standardized rubric. In addition to general verbal and non-verbal communication skills, this rubric also assessed empathy, establishing trust, and professionalism. Due to the COVID-19 pandemic, and subsequent halting of in-person learning, the 2020 cumulative OSCE was administered online using Zoom. This was the only modification made to the 2020 OSCE; all materials, procedures, and grading remained the same between the two cohorts. 

For both examinations, differences between the cohorts for each sample were examined using non-parametric Mann Whitney U Tests. Effect sizes were calculated to provide a description of significant findings. We used two different exams as different variables were assessed in each exam to strengthen the validity of the virtual examination process and related outcomes. Variables of interest included overall performance for both examinations, demonstration of empathy, verbal and non-verbal communication, establishing trust, patient friendly language, organization and professionalism. The project was determined to be exempt by the Colorado Multiple Institutional Review Board. 

## 3. Results

Examination performance data for the 2019 (*n* = 144) and 2020 (*n* = 106) cohorts were included in the analyses. For the PCC2 assessment (Table 1), the 2020 (*n* = 104) cohort scored significantly higher than the 2019 (*n* = 134) cohort on all variables including overall score. For the OSCE (Table 2), the 2020 cohort scored significantly higher on the trust variable. No other significant differences were found between groups on the OSCE. 

## 4. Discussion

Data from the PCC2 Patient Counseling OSCE showed student scores on all variables were statistically significantly higher in the 2020, virtual cohort. Student scores on the cumulative OSCE were statistically significantly higher in the global feedback variable of establishing trust. Students performed similarly between the in-person (2019) and online (2020) OSCE on all other variables. While there were statistically significant differences between 2019 and 2020 scores, with higher scores in the 2020 (virtual) groups, median scores and interquartile ranges were similar across all variables, thus effect size was calculated for significant findings. The PCC2 variable related to establishing trust barely crossed the threshold to be described as a medium size effect. All other comparisons of these effect sizes can be described as small. Anecdotally, course directors mention the possibility of evaluators unconsciously grading more leniently in light of challenges related to the COVID-19 pandemic. In addition, due to limitations with virtual proctoring, there is a risk for decreased exam integrity. As 2019 was also our first year for the cumulative OSCE, it was primarily used to pilot the process and obtain preliminary data, and students were not penalized for poor performance. Brief examination of frequency tables do support this notion as there were more scores at the lower end of the scale in 2019 than in 2020. 

With the recent transition to remote assessments and the telehealth practice environment, there is increasing concern that the patient experience is lacking because communication strategies that build trust and rapport, such as eye-contact, professionalism and displaying empathy, may be absent or deficient in the virtual environment. This comparison of two different patient communication assessments performed in person and virtually, demonstrates that these skills were not lost in the virtual realm. 

Though these virtual assessments were completed successfully via Zoom, there were many lessons learned to improve our ability to smoothly create and run these virtual OSCE’s. To start, training the evaluators, faculty and SPs should be done using live, online training sessions and include mock run through sessions. Outside programs, such as Microsoft Teams (Microsoft Teams version Microsoft Teams in Office 365), should be utilized for external communication regarding exam questions and alerting the hosts of technical issues. Mandatory practice assessments that mimicked the exact format of the assessment proved to be critical in the efficiency in which the actual assessments were run. These practice sessions allowed students, faculty and SPs to work through potential issues, technology and logistics in a low-stakes environment. Practice assessments also improved consistency in grading as questions regarding the rubric and student performance were discussed prior to the exam. In a recent article detailing tips for conducting an OSCE in a virtual environment for medical students, Hopwood and colleagues recommend extra training including completing a full-run through of the OSCE the day prior [6]. 

Other methods to explore for online assessment, and to give students more practice in communication skills in the virtual setting, include using programs with virtual patients. The use of virtual simulated patients, such as computer-based “patients” with standardized responses, is an emerging method used to train clinical and communication skills in the virtual environment [7]. This method is a feasible way to prepare students for communication-based OSCEs, and it has demonstrated ability to improve communication-related skills such as enhancing ability to display empathy in a patient encounter [8,9]. However, results are mixed regarding if the level of empathy demonstrated to a virtual patient is more or less than that expressed to a human SP [10]. Simulated telehealth patient care through electronic messaging has also been integrated into the assessment of a self-care course, where students communicated with SPs over email [11]. 

We recommend using SPs for virtual assessments to provide a more real experience for students. The use of SPs for OSCEs is well-supported in the literature. In a study by Cho et al. regarding faculty and student perception of the use of SPs for OSCEs, the majority of students reported that SPs portrayed patients more realistically and created a more comfortable environment for patient communications assessments than faculty and staff. The majority of students also reported feeling more confident in their communication and ability to make recommendations when SPs were used [12]. In addition, Gillette et al. found that incorporating SPs within a communication course led to improved scores and increased first-time pass rates on communication assessments [13]. 

Having proctors or hosts is integral to running a smooth assessment [6]. Proctoring or hosting virtual based assessments requires the ability to orient students, provide instructions, place students in appropriate breakout rooms and handle technology related issues. Occasional schedule delays are inevitable in the virtual setting due to technology related issues, and having extra timeslots built in the schedule can provide make-up opportunities. We recommend having a backup room and backup facilitator and/or SP for students who are having technology related issues. We also recommend having at least two proctors for these exams, which allows one to assign student rooms, while the other handles technology related issues.

Lastly, maintaining academic integrity in the virtual environment is a challenge that has required creativity and adaptability to find solutions. We use room and work place scans to ensure there are no notes, rubrics or electronic devices present. We also have students share their screens to ensure they do not have documents on their screen that are prohibited on the assessment. We require students to use gooseneck cameras for online examinations so we can see their computer screen since this area is not seen with typical laptop cameras during a room scan. We have the SP or facilitator record the session, including the initial room scans, so the videos can be reviewed at a later time if there are questions regarding academic integrity. These videos may be released to students after the completion of the assessment for self-reflection and learning. Assessing students on skill rather than knowledge lends itself very well to virtual assessments where there is concern for the maintenance of academic integrity, as inappropriate sharing of assessment-related information is irrelevant when students are assessed based on skill demonstration. Similar to strategies described by Lucas and colleagues, we also maximize academic integrity by requesting students to present their identification cards upon entry to the online meeting room, requiring that students have their microphone and video on at all times, and providing different scenarios or cases part way through assessments [14].

## 5. Conclusions

Conducting OSCEs including communication skills is possible in a virtual format as supported by the results seen at SSPPS. Students completing the virtual OSCE performed minimally as well as students delivering in-person patient counseling and gathering in-person medical histories. Communications-based OSCEs delivered in a virtual manner can serve as a bridge for educational programs as they move towards emphasizing telehealth skills, which as recognized as critical in the future of pharmacy. Experiences at SSPPS revealed virtual assessment is not a detriment to the learner, and that trust and empathy can be developed and maintained in the virtual environment. SSPPS is likely to continue using a hybrid of virtual and in person assessments going forward. We implore other programs to explore virtual methods, if able, to conduct skill assessments over eliminating skill assessments with limitations to in-person assessments during the pandemic.

## Figures and Tables

**Table 1:** Examination Performance Data, 2019 and 2020, Patient Centered Communication 2 (PCC2) Evaluation.

	2019		2020			
Variable	Median	Range	Median	Range	*p* Value	Effect Size *
Overall Score (Percent)	96.47	36.47	99.00	23.00	0.000	−0.29
Establishing a Trusting Relationship	10.00	7.00	10.00	7.00	0.000	−0.32
Effective Verbal and Non-Verbal Communication	10.00	7.00	10.00	7.00	0.001	−0.21
Provided Patient Friendly Education	10.00	7.00	10.00	7.00	0.026	−0.14
Organizing the Encounter	10.00	7.00	10.00	3.00	0.044	−0.13

* Effect size descriptors: 0.10–0.30 small; 0.30–0.50 medium; ≥0.50 large.

**Table 2:** Examination Performance Data, 2019 and 2020, Observed Structured Clinical Examination (OSCE).

	2019		2020			
Variable	Median	Range	Median	Range	*p* Value	Effect Size *
Demonstrates Empathy	4.00	4.00	4.00	4.00	0.245	
Appropriate Non-Verbal Comm.	4.00	4.00	4.00	4.00	0.259	
Professionalism	4.00	4.00	4.00	4.00	0.750	
Global Feedback: Establishing Trust	4.00	4.00	4.00	4.00	0.015	−0.15
Total Variable Score	16.00	10.00	16.00	16.00	0.039	−0.13

* Effect size descriptors: 0.10–0.30 small; 0.30–0.50 medium; ≥0.50 large.

## Data Availability

The data presented in this study are available on request from the corresponding author.
